# Motor improvement of remote programming in patients with Parkinson's disease after deep brain stimulation: a 1-year follow-up

**DOI:** 10.3389/fneur.2024.1398929

**Published:** 2024-06-19

**Authors:** Xiaonan Wan, Chengcheng Duan, Zhengyu Lin, Zhitong Zeng, Chencheng Zhang, Dianyou Li

**Affiliations:** ^1^Department of Neurosurgery, Center for Functional Neurosurgery, Ruijin Hospital Affiliated to Shanghai Jiaotong University School of Medicine, Shanghai, China; ^2^Clinical Neuroscience Center, Ruijin Hospital Luwan Branch, Shanghai Jiao Tong University School of Medicine, Shanghai, China; ^3^Shanghai Research Center for Brain Science and Brain-Inspired Technology, Shanghai, China

**Keywords:** Parkinson's disease, deep brain stimulation, remote programming, telemedicine, motor symptoms

## Abstract

**Background:**

Remote programming (RP) is an emerging technology that enables the adjustment of implantable pulse generators (IPGs) via the Internet for people with Parkinson's disease (PwPD) who have undergone deep brain stimulation (DBS). Previous studies have not comprehensively explored the effectiveness of RP in managing motor symptoms, often omitting assessments such as the rigidity and retropulsion tests during the follow-up. This study evaluates the comprehensive improvements in motor performance and the potential cost benefits of RP for PwPD with DBS.

**Methods:**

A retrospective analysis was conducted on two groups of patients—those who received RP and those who received standard programming (SP). Clinical outcomes including motor improvement, quality of life, and daily levodopa dosage were compared between the groups during a 12 (± 3)-month in-clinic follow-up.

**Results:**

A total of 44 patients were included in the study, with 18 in the RP group and 26 in the SP group. No significant differences were observed in the frequency of programming sessions or clinical outcomes between the groups (p > 0.05). However, the RP group experienced significantly lower costs per programming session than the SP group (*p* < 0.05), despite patients in the former group living further from our center (*p* < 0.05).

**Conclusions:**

Our findings suggest that RP could significantly reduce the costs of programming for PwPD with DBS, especially without compromising the effectiveness of treatment across all motor symptoms in the short term.

## Introduction

Deep brain stimulation (DBS) is a proven surgical treatment modality for people with Parkinson's disease (PwPD) who do not respond adequately to oral medications, achieving its best results through precise programming of stimulation parameters tailored to symptom fluctuations (1–3). However, the need for patients and their caregivers to travel to specialized centers for standard programming (SP) places significant economic burdens on them (4). Remote programming (RP) enables clinicians to adjust stimulation parameters via the Internet, which is recognized as both safe and effective (5, 6), thereby enhancing postoperative management of PwPD with DBS implants.

Rigidity, a typically used clinical response in SP (7), could not be directly assessed through videoconferencing in RP. It remains uncertain if the omission of rigidity tests in RP could be offset by the assessment of other symptoms such as bradykinesia and tremors. This uncertainty is particularly notable owing to the fact that most previous studies on RP have excluded the rigidity and pullback tests from their motor evaluations (8, 9). Additionally, studies by Chen et al. (10) and Nie et al. (6) compared patients who received RP with those who received SP, but the actual motor assessments were not conducted on the RP group.

This retrospective cohort study aims to evaluate the superior effect of RP in comprehensive motor symptoms by using the complete Movement Disorder Society (MDS)-Unified Parkinson's Disease Rating Scale part III (UPDRS III) scores and to assess the associated costs of programming sessions. Our findings are expected to support the adoption of RP as a potential method for PwPD who face significant travel cost burdens related to post-DBS programming.

## Methods

### Participants

We recruited PwPD who underwent DBS at our center from January 2018 to December 2022. Patients who met the following inclusion criteria were enrolled: (1) those who received bilateral subthalamic nucleus (STN) DBS and subsequent programming at our center; (2) those who could be followed up within 12 ± 3 months after DBS implantation; and (3) those who had a programming history where at least 65% of sessions were conducted via RP, or all sessions were completed via SP, leading to being them assigned into the RP or SP group, respectively (Supplementary material S1).

The IPGs used in this study were from three manufacturers: PINS (11), SceneRay (12), and Medtronic. Notably, the Medtronic IPGs were only included in the SP group as they lack RP capabilities.

### Cost model and caregiver burden questionnaire

A comprehensive cost model was developed to analyze patient expenses, including transportation costs, lost working time, and fees per programming session. All SPs were carried out in an impatient clinic. Caregiver burden was assessed using a specialized questionnaire during the follow-up for the caregivers, which evaluated the number of required caregivers and lost working time per programming session. Additional details are provided in Supplementary materials S2, S3. The cost was converted to US dollars (USD), based on the exchange rate of 1 USD ≈ 7.2445 RMB.

### Data collection

The UPDRS III scores were recorded at baseline (during a levodopa challenge test) and at an in-clinic follow-up (with active DBS stimulation and medications washed out) (13, 14). Assessments were conducted and scored by two independent, blinded raters at our center. Additional data collected included the levodopa equivalent daily dose (LEDD) and the 8-item Parkinson's disease questionnaire (PDQ-8) scores, where higher scores indicate poorer quality of life (15). Detailed data on programming sessions and patient demographics were extracted from the RP systems (PINS, APP, “JiayiYoupin” and SceneRay, APP, and “Jingyun Internet Hospital”) and our Electronic Medical Record System. Cybersecurity details have been documented elsewhere (16).

### Programming schedule

Postoperative CT scans were conducted within 48 h following the DBS surgery to confirm the placement of electrodes (17), and IPGs were programmed to deliver narrow bipolar stimulation at ~1.0 V (130 Hz; 60 μs) as a temporary stimulation before patient discharge. The initial programming was scheduled 1 month post-surgery after local edema subsided. Subsequently, patients were recommended to undergo four to five additional programming sessions within the following 6 months (2). However, the interval and method of each programming session varied among patients, which was usually scheduled based on their own feelings and individual preferences. All RP and SP sessions were conducted by an experienced and trained physician, Dr. D.L., thus minimizing potential variability in programming quality.

### Remote programming procedure

The basic procedures of the RP have been outlined in our recent study on obsessive compulsive disorder (18). The process begins once a patient successfully schedules an appointment. RP service staff then assess the hardware environment, ensuring the network's stability, the clarity of sound and video, and the availability of sufficient space to perform various actions, including standing from a seated position and walking.

During each RP session, the physician checks the electrode impedance of DBS and confirms the placement of electrodes using fused images from postoperative CT and preoperative MRI scans. PwPD are instructed to sit in a straight-backed chair with arms resting on the armrest and feet flat on the floor and perform specific actions as directed by the physician to evaluate the stimulation effects. Adjustments to the parameters are made based on motor performance and electrode positioning. Although rigidity cannot be assessed through RP, visually assessable symptoms such as tremors and bradykinesia are monitored to titrate the amplitude. The assessment includes specific items from UPDRS-III (items 3.4–3.8 and 3.14–3.18), which are consistently used in both RP and SP sessions. Furthermore, stimulation-induced side effects such as speech impairment, dyskinesia, and facial pulling were either observed directly or reported by patients, aiding in determining the therapeutic window. Each amplitude increment is monitored for up to 5 min, varying with individual responses. When a new parameter is set, the previous setting is preserved to ensure that it can be restored if the new adjustment proves to be intolerable.

### Statistical analysis

Data are expressed as counts (percentages) or medians (interquartile range, IQR). A *p*-value of > 0.05 was used as the non-inferiority margin. Categorical data were analyzed using Fisher's exact test, and continuous variables were assessed using either the t-test or Wilcoxon rank-sum test, depending on the distribution. All tests were two-sided, with significance set at a *p* < 0.05. Data were managed and analyzed using Excel (Microsoft, San Jose, CA, USA) and SAS 9.4 (SAS Institute Inc, Cary, NC, USA).

### Ethical consideration

Written informed consent for the research was obtained from all participants prior to their follow-up. The study protocol was approved by the Ethics Committee of the Ruijin Hospital [Clinical Ethics Review (2023) No. 231] and adhered to the principles of the Declaration of Helsinki.

## Results

### Population characteristics

**Figure 1:**
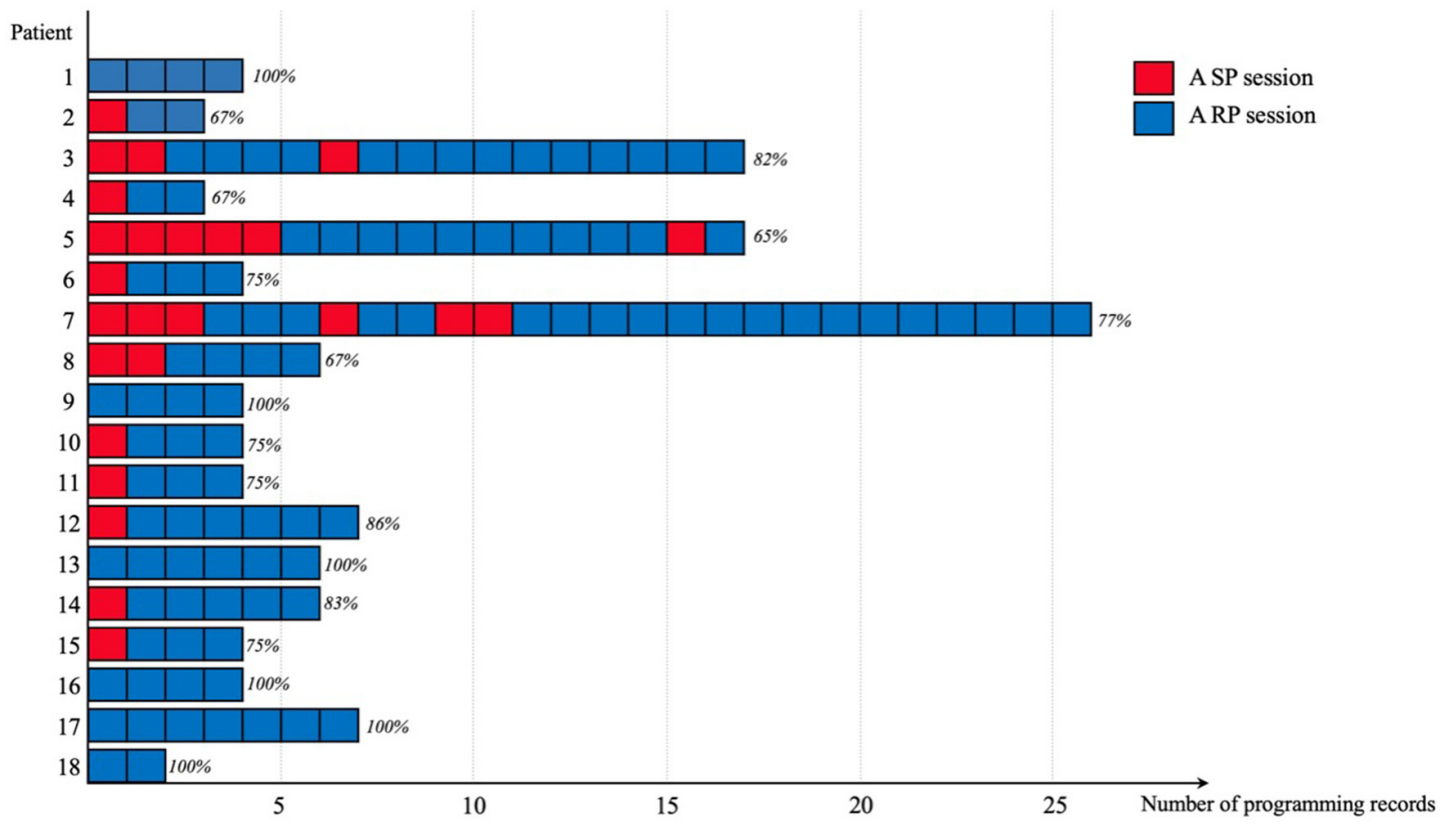
Distribution of programming records among patients in the RP group. Each bar represents a programming record, categorized into sessions of RP (remote programming, shown in blue) and SP (standard programming, shown in red). The percentages above each bar indicate the proportion of RP sessions in relation to the total number of programming sessions for each patient.

This study included 44 patients, categorized into 18 patients in the remote programming (RP) group and 26 controls in the standard programming (SP) group. The programming history of the RP group, as shown in Figure 1, included 128 programming sessions, 80% (103/128) of which were conducted via RP. The two groups were comparable in most baseline characteristics, except for living distance from our center (*p* < 0.05, Table 1). The UPDRS III scores and LEDD data were collected from all patients, while PDQ-8 scores were available for 27 patients.

**Table 1:** Characteristics of the included patients in the baseline.

**Item**	**RP**		**SP**		
	**N**	**Median (IQR)**	**N**	**Median (IQR)**	**p**
Age	18	64 (52–67)	26	64 (60–69)	0.282
Gender (woman)	7	-	11	-	1.000
Disease duration (years)	18	9 (6–13)	26	10 (6–13)	0.948
Follow-up (months)	18	14 (11–14)	26	13 (12–16)	0.380
Distance (km)	18	423 (161–511)	26	31 (14–151)	0.002^*^
Total UPDRS- III^a^	18	53 (45–66)	26	59 (47–64)	0.871
- Rigidity		14 (10–16)		13 (11–15)	0.822
- Tremor		8 (3–16)		10 (3–15)	1.000
- Bradykinesia		23 (22–28)		22 (21–28)	0.957
- Axial symptoms		9 (6–13)		10 (7–13)	0.581
LR (%)	18	52 (44–58)	26	46 (35–54)	0.084
LEDD	18	726 (525–900)	26	600 (450–850)	0.665
PDQ-8	13	13 (8–16)	14	12 (7–15)	0.559

IQR, interquartile range; UPDRS-III, The Movement Disorder Society Unified Parkinson's Disease Rating Scale—part III; LR, levodopa responsiveness in the levodopa challenge test; LEDD, levodopa equivalent daily dose; PDQ-8, The 8-item Parkinson's Disease Questionnaire. ^*a*^ Motor performance was recorded for patients when medication was washed out before the surgery. ^*^A p < 0.05 was considered statistically significant.

### Clinical outcomes and programming burden

Significant improvements were observed in UPDRS III scores, PDQ-8 scores, and LEDD from baseline to follow-up in all patients (*p* < 0.05). However, the rates of change in these clinical outcomes did not significantly differ between the two groups (Table 2). There were no reports of severe adverse events or complications.

**Table 2:** Changes of clinical outcomes and travel cost between the two groups in the follow-up.

**Item**		**RP**		**SP**		
		**N**	**Median (IQR)**	**N**	**Median (IQR)**	**p**
Changes (%)^a^	Total UPDRS- III^b^	18	45 (32–52)	26	43 (26–53)	0.858
	- Rigidity		49 (19–88)		44 (23–71)	0.908
	- Tremor		64 (50–95)		90 (63–100)	0.139
	- Bradykinesia		37 (15–55)		33 (6–50)	0.642
	- Axial symptoms		19 (0–44)		25 (7–36)	0.774
	LEDD	18	43 (24–58)	26	47 (33–64)	0.281
	PDQ-8	13	39 (13–50)	14	31 (0–71)	0.828
Travel cost (USD)^c^		18	46 (28–65)	26	79 (41–123)	0.010^*^
Total sessions		18	4 (4–7)	26	6 (3–8)	0.809

IQR, interquartile range; UPDRS-III, The Movement Disorder Society Unified Parkinson's Disease Rating Scale—part III; LEDD, levodopa equivalent daily dose; PDQ-8, The 8-item Parkinson's Disease Questionnaire; USD, US dollar. ^*a*^Rate of changes was calculated using the formula {100% ^*^ (Outcome_baseline_ – Outcome_follow-up_)/Outcome_baseline_}. ^*b*^Motor performance was recorded during medication washout periods at both baseline and follow-up assessments. ^*c*^Average travel cost per programming session was calculated using the formula (Total travel cost / total times of programming sessions). ^*^A p < 0.05 is considered statistically significant.

**Figure 2:**
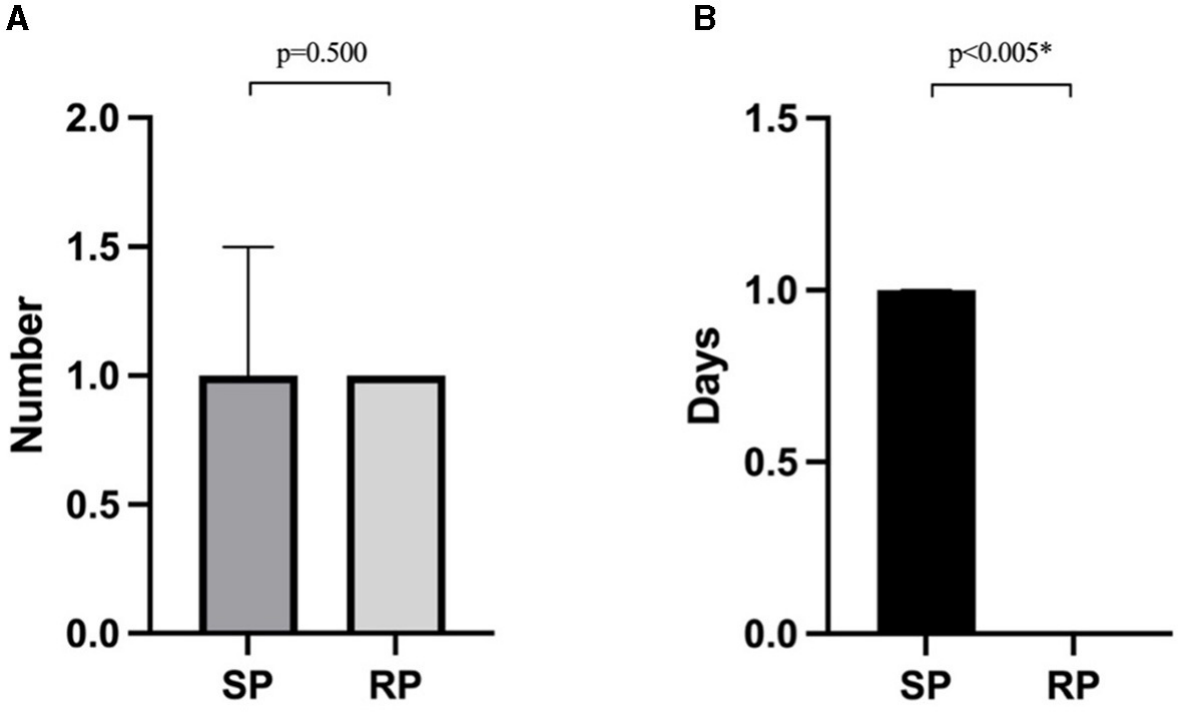
Caregiver burdens associated with two programming methods in the RP group: **(A)** the number of caregivers required for each programming session; **(B)** the lost working time for patients or their caregivers incurred per programming session. *A *p* < 0.05 is considered statistically significant.

The frequency of programming sessions did not differ significantly between the groups, with the RP group averaging four sessions (IQR: 4–7) and the SP group averaging six sessions (IQR: 3–8) (Table 2). The average cost per programming session was lower in the RP group [with USD$46 (IQR: 28–65)] than in the SP group [with USD$79 (IQR: 41–123)] (*p* < 0.05, Table 2). In the RP group, the burden on caregivers showed no significant differences in the number of caregivers needed between the two methods, but the lost working time for each RP session was significantly reduced (*p* < 0.05, Figure 2).

## Discussion

This study compared two groups of PwPD who received either RP or SP to evaluate the clinical effectiveness of RP. Key findings include the following: (1) RP achieved motor improvements comparable to those of SP during the 1-year postoperative period, demonstrating the feasibility of using visually assessable symptoms as a primary clinical response in RP and (2) RP alleviated the economic burden for PwPD with DBS, making it a recommended option for those challenged by frequent in-clinic visits.

Although rigidity is a common clinical response in SP, evaluating the effects of stimulation requires a systematic review of multiple factors, not just a single clinical sign (7). In cases where rigidity is not pronounced, symptoms such as tremors and bradykinesia are evaluated, though responses to these may be slower and may vary among patients (19–21). In our study, we observed each increase in amplitude for up to 5 min to thoroughly assess the full response to stimulation (21). The duration of RP also varied based on response times in PwPD. Although rigidity could not be evaluated in RP, our findings indicate that motor improvement in RP was not inferior to that observed in SP.

During the COVID-19 lockdown, many patients attempted remote programming (RP) for the first time, leading to a significant increase in demand (22). This study, conducted without imposing any of the quarantine restrictions, observed that eight patients in the RP group chose to continue using RP after their initial experience, demonstrating a growing familiarity with and a preference for this method. There was no significant difference in the number of programming sessions between the two groups. However, three patients in the RP group had experienced significantly more programming sessions (outliers: Patients 3, 5, and 7; see Supplementary material S4). Patient 3 developed stimulation-induced dyskinesias, which required smaller increases in stimulation and a longer interval between assessments; Patient 5 and Patient 7 sought additional programming due to perceived inadequate symptom control, primarily related to gait impairments. Despite these challenges, their motor improvement was comparable to the average (rate of change: 37%, 62%, and 41%, respectively).

Six patients successfully completed the initial programming using the RP. According to the expert consensus, initial programming required more detailed contact screening and parameter titration than the following programming (3, 17). A previous study reported 23 patients who completed all initial and follow-up programming sessions by RP (10). However, considering the omitted rigidity test and physician variants, the safety and feasibility of initial programming via RP require further exploration.

This finding is consistent with those of previous research, which has documented significant improvements in motor symptoms and quality of life through RP (9, 10). Additionally, although not statistically significant, we noted an increased reduction in conservative management in the RP group compared to the SP group, similar to the findings reported by Chen et al. (10). This variation may reflect differing treatment approaches among physicians.

RP offers significant advantages in terms of flexibility and cost-effectiveness, especially for patients who live far from specialized centers. According to our data, the RP group incurred lower costs over 1 year, despite living farther from our center. This cost efficiency also benefited caregivers, who reported reduced lost working time compared to the SP group. However, in the RP group, the same number of caregivers was required as the SP group, which could likely be attributed to the elderly patient demographics, who required assistance setting up the videoconferencing equipment.

As DBS has been increasingly used across various diseases, the labor-intensive postoperative management is increasingly burdensome for both medical staff and patients. Feedback from patients was indispensable in the programming session. For physicians, auxiliary technologies such as closed-loop stimulation (23) and visualization stimulation (24) are being developed to optimize the workflow. For PwPD, various wearable devices are available to monitor clinical features such as tremors, dyskinesia, and freezing of gait (25–27). Continuous feedback from these devices could help physicians adjust stimulation more suitably to individual daily variations in symptoms and activities. Moreover, conducting assessments via RP at home is increasingly favored by PwPD due to the comfort and convenience of the familiar environment (28). The integration of RP with newer technologies presents a significant potential for advancing treatment methods.

This study is subject to several limitations. (1) The inclusion criterion for the RP group was based on “over 65% of programming sessions completed through RP.” A more stringent criterion might have provided a clearer validation of the impact of RP. (2) As a single-center study, the findings may be affected by the small and uneven sample sizes. Incomplete records for some patients may also compromise the reliability of the QoL improvement outcomes. These results should be interpreted with caution, particularly regarding their applicability to other settings. Additionally, the experience of the programming physicians could impact the outcomes. Future studies across multiple centers with larger and more balanced groups would enhance the statistical robustness. (3) The follow-up duration for the participants was relatively short. Over the long term, complex symptoms such as gait disturbances and swallowing difficulties may emerge (29). These issues require thorough assessments, for which SP might be more appropriate.

## Conclusion

This study demonstrates that RP could provide better motor improvements compared to SP among PwPD undergoing STN DBS while also reducing the logistical burdens associated with travel to programming sessions. The efficacy and convenience of RP are crucial for enhancing DBS management, particularly for those challenged by repeated in-clinic visits. While the findings advocate for the increased integration of RP into standard postoperative care, further research is necessary to explore the long-term benefits and the feasibility of initial programming via RP.

## Data availability statement

The raw data supporting the conclusions of this article will be made available by the authors, without undue reservation.

## Ethics statement

The studies involving humans were approved by the Ruijin Hospital Ethics Committee. The studies were conducted in accordance with the local legislation and institutional requirements. The participants provided their written informed consent to participate in this study.

## Author contributions

XW: Writing – original draft, Writing – review & editing. CD: Writing – original draft. ZL: Writing – review & editing. ZZ: Writing – review & editing. CZ: Writing – review & editing. DL: Writing – review & editing.
