# Supplementary material for: Motor improvement of remote programming in patients with Parkinson's disease after deep brain stimulation: a 1-year follow-up
Source: Front Neurol. 2024 Jun 19;15:1398929. doi: 10.3389/fneur.2024.1398929 (PMC11220248; doi:10.3389/fneur.2024.1398929)
Supplement: Supplementary file 1 [file Table_1.DOCX]

# **Supplementary material**


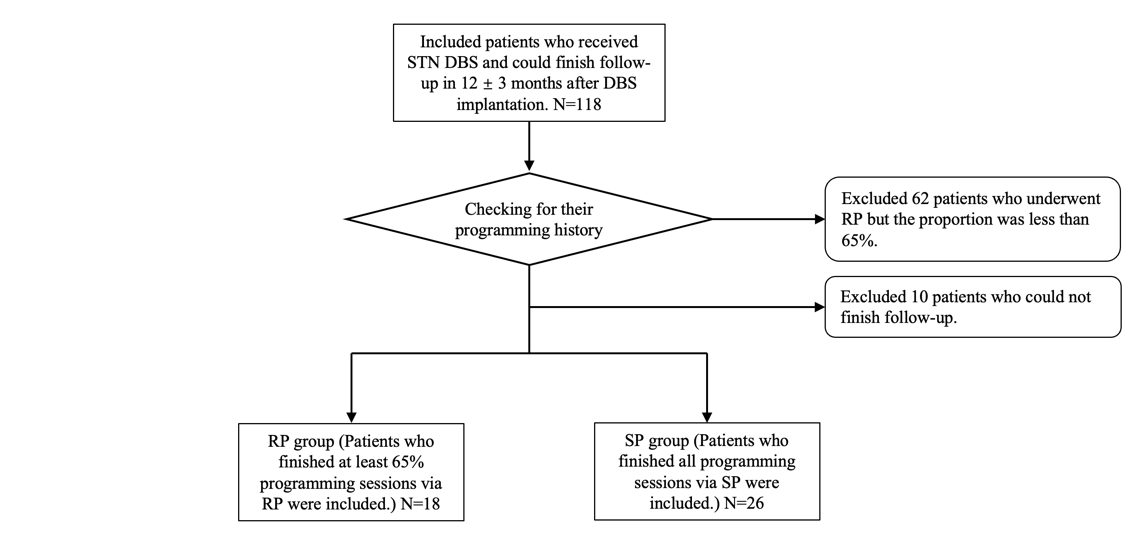


S1. Flowchart illustrating the patient selection process for this study. Patients were categorized into two groups: the RP group (N=18), who completed at least 65% of their sessions, and the SP group (N=26), who completed all sessions as per standard programming."

S2. Variables in the cost model of this study.

| items | Cost |
| --- | --- |
| Travel fare, TF (Yuan/ km) | Train: 0.46 |
|  | Taxi: 2.7 |
| Lost income for in-person visits, LI (Yuan/ day) | 101.5 |
| Registration fee, RF (Yuan) | 100 |
| Accommodation, AC (Yuan) | 300 |
| Cost of each SP (Yuan) | TF*4+LI+RF+AC |
| Fee of each RP (Yuan) | 200 |

Abbreviation: Yuan, Chinese yuan. The cost was converted to US dollars, based on an exchange rate of 1 USD ≈ 7.2445 RMB.

S3. Caregiver burden questionnaire

1. How many caregivers do you need to accompany each standard programming?

A. I can do it by myself.

B. One

C. Two

D. Three and above: ___

2. How many days does your caregiver take off work for each standard programming?

A. No need to take off work.

B. 1/2 day

C. One day

D. Two days and above: ___

3. How many caregivers do you need to accompany each remote programming?

A. I can do it by myself.

B. One

C. Two

D. Three and above: ___

4. How many days does your caregiver take off work for each remote programming?

A. No need to take off work.

B. 1/2 day

C. One day

D. Two days and above: ___


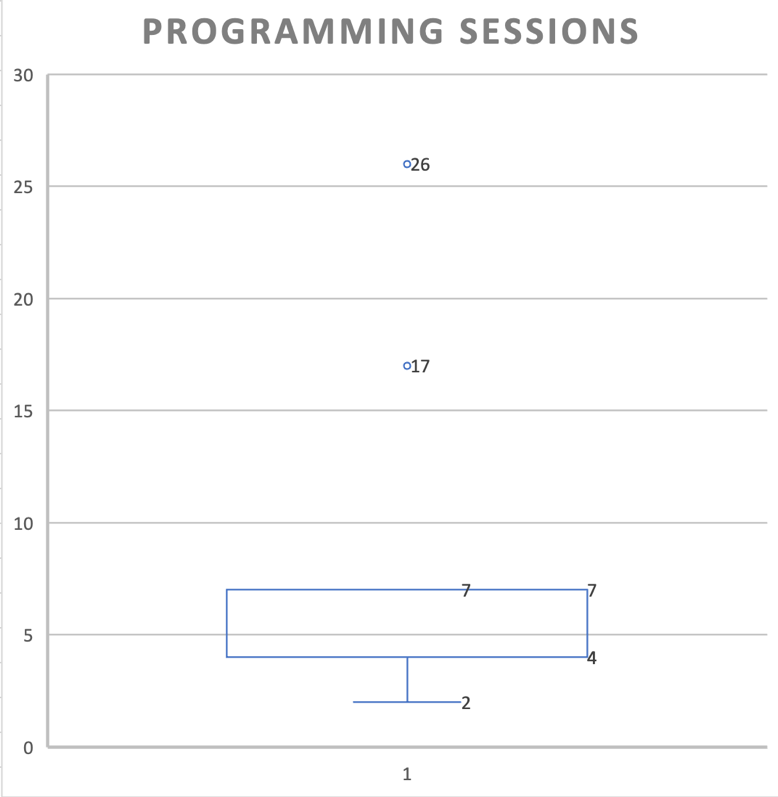


S4. Box and whisker plot of the total programming sessions in the RP group.
